# Control and maintenance of mammalian cell size: Response

**DOI:** 10.1186/1471-2121-5-36

**Published:** 2004-09-30

**Authors:** Ian Conlon, Martin Raff

**Affiliations:** 1 MRC Laboratory for Molecular Cell Biology and Cell Biology Unit, University College London, London WC1E 6BT, UK; 2 Great Minster House, 77 Marsham Street, London SW1P 4DR, UK

## Abstract

A response to Cooper S: **Control and maintenance of mammalian cell size. ***BMC Cell Biol *2004, **5**:35

Stephen Cooper was kind enough to send to us an original draft of his paper that now appears in *BMC Cell Biology *[1]. Although we have exchanged a number of e-mails with him in attempt to clarify points of confusion and disagreement, he continues to tilt at windmills and attack straw men. This is discouraging.

His paper claims to be an analysis of our paper that was published in *Journal of Biology *last year [2]. Unfortunately, in much of his paper, he challenges our answers to questions that we did not address, conclusions that we did not draw, and arguments that we did not make. There are too many examples of this to deal with them all.

One problem is semantic. He uses the term "cell growth" ambiguously, to mean both cell enlargement and cell number increase, which is confusing when discussing both cell growth and cell proliferation. In the Background, for example, he writes "How do cells maintain a constant cell size and cell size distribution during extended cell growth?".

There is more important confusion in the phrase "linear growth". He uses it to mean the addition of an equal amount of mass at each stage of the cell cycle, and he claims that we use it in our paper in the same way. In our paper, however, we clearly defined linear growth to describe our observation that Schwann cells, blocked in S phase with aphidicolin, added a constant amount of volume and mass per cell over time, independent of their size. This confusion leads him to claim erroneously, in his Abstract and elsewhere, that we proposed that mammalian cells grow linearly during the division cycle; we do not believe this, and we did not test it or discuss it in our paper. Much of his paper is based on the premise that we were trying to understand how cell growth changes through the cell cycle. In fact, we have never addressed this question, in the paper or elsewhere. For this reason, much of Cooper's paper is not relevant to ours.

Cooper criticizes individual experiments in our paper, but this too is almost always based on unnecessary misunderstanding. He accuses us, for example, of "an egregious error" in studying protein synthesis in Schwann cells that were not synchronized and therefore in all phases of the cell cycle. In fact, however, the cells were all arrested at the start of S phase with aphidicolin, as pointed out in both the text and figure legend.

Despite its length, Cooper's paper never comes to grips with either the two main findings in our paper or the points of the experiments described in it. Unlike yeast cells (*S. pombe*) blocked in S phase by a mutation, which grow faster as they enlarge [3], we found that Schwann cells blocked in S phase with aphidicolin continue to grow at the same rate as they enlarge, adding a constant amount of volume and protein each day, independent of their size, We argued, as have others [4], that if big and little cells grow at the same rate (at the same point in the cell cycle), they do not need a cell-size checkpoint to maintain a constant distribution of sizes as they proliferate, unlike the situation for yeast cells. A second important difference from yeast cells that we found was that Schwann cells shifted from serum-free medium to serum-containing medium took 5–6 divisions and more than a week to attain the larger size of cells continuously proliferating in serum. This is what one would predict for cells that do not have a cell-size checkpoint and where little cells grow at the same rate as big cells at the same point in the cell cycle [2,4]. By contrast, when similar shift-up experiments are performed with yeast cells, the cells attain their new larger size within one cell cycle when shifted from a nutrient-poor culture medium to a richer medium [5]. We concluded that, if Schwann cells have cell-size checkpoints, they are very different form those that operate in yeast cells.

Cooper also ignores our earlier findings that Schwann cell size at division depends simply on how fast the cells are growing and how fast they progress through the cell cycle and that both of these rates depend on the concentrations of extracellular signals that can regulate the two rates independently [6]. We found that GGF, for example, stimulated cell-cycle progression in these cells but not cell growth, whereas IGF-1 stimulated cell growth and synergized with GGF to stimulate cell-cycle progression. When IGF-1 concentration (and therefore cell growth) was held constant, an increase in the concentration of GGF drove the cells through the cycle faster; with less time to grow, the cells were smaller in high GGF compared to low GGF, at all stages of the cycle. These findings do not fit easily with Cooper's model that cell mass is the driving force of the cell cycle in all cells.

Cooper's model for how cell growth and cell division can be coordinated is one version of a cell-size checkpoint model, in which progression through the cell cycle is somehow linked to cell size. Such models have been widely accepted in the cell-cycle field to explain how proliferating cells maintain their appropriate size over time [7]. Whereas the evidence for cell-size checkpoints in single-cell organisms is strong, the evidence for them in animal cells is weak, despite Cooper's arguments to the contrary. Our studies suggest that cultured Schwann cells (and we suspect many other animal cells) do not need, and probably do not have, such cell-size checkpoints to coordinate their growth and division. This difference between single-cell organisms and animal cells is not surprising given their very different life styles: in bacteria and yeasts, cell growth and proliferation are controlled mainly by nutrients, whereas in animals, they are controlled mainly by signals from other cells.
